# Investigating the association between diabetes and mental health: A train-and-test approach

**DOI:** 10.3389/fpsyt.2022.1044714

**Published:** 2022-12-19

**Authors:** Weixi Kang

**Affiliations:** Department of Brain Sciences, Imperial College London, London, United Kingdom

**Keywords:** diabetes, mental health, depression, anxiety, social dysfunction, anhedonia, loss of confidence

## Abstract

Diabetes is a chronic health condition that affects how the body turns food into energy. Research has demonstrated a relationship between diabetes and various mental health issues, which include psychiatric disorders and other problems that are specific for people living with diabetes. Although previous studies have shed light on the associations between diabetes and various types of mental health issues with a focus on depression and anxiety, much less is known about how diabetes is associated with other dimensions of mental health such as social dysfunction and anhedonia and loss of confidence in a large nationally representative survey from the United Kingdom. The aim of the current study is to replicate the factor structure of the GHQ-12 and investigate how diabetes is related to general mental health and dimensions of mental health. By adopting a train-and-test approach to data from the UKHLS including 2,255 diabetes patients and 14,585 age and sex-matched participants who indicated that they were not clinically diagnosed with diabetes, the current study found that hypotheses are well-supported by the results.

## Introduction

Diabetes is a demanding chronic disease for both individuals and their families (1). Diabetes is characterized by elevated glucose in the blood. Type 1 diabetes refers to the fact that the pancreas produces no insulin whereas type 2 diabetes refers to the case where the pancreas produces an insufficient amount of insulin. Research has demonstrated a relationship between diabetes and various mental health issues, which include psychiatric disorders and other problems that are specific to people living with diabetes (2). There are some terms that are used to characterize the mental health problem associated with diabetes. For instance, “diabetes distress” refers to the negative emotions and burden of self-management in people with diabetes, which is used to describe “the despondency and emotional turmoil specifically related to living with diabetes, in particular the need for continual monitoring and treatment, persistent concerns about complications, and the potential erosion of personal and professional relationships (2–4). “Psychological insulin resistance” refers to the refusal or reluctance to accept insulin therapy, which can delay the start of the treatment for a period of time (5). Fear of hypoglycemia is another common concern in diabetes. Understanding the association between diabetes and mental health is important because psychiatric and diabetes-specific psychosocial issues are related to reduced participation in self-management activities that can decrease the quality of life. Moreover, psychiatric disorders in diabetes patients increase the risk of diabetes complications and early mortality (6).

Indeed, previous studies have found that diabetes is associated with major depressive disorder [e.g., (7–10)], anxiety [e.g., (11, 12)], bipolar disorders [e.g., (13, 14)], schizophrenia (15), personality disorders [e.g., (16, 17)], stress, trauma, abuse and neglect (18, 19), eating disorders (20, 21), and sleep issues [e.g., (22)]. Moreover, the associations between diabetes and mental health could be totally bi-directional [e.g., (10)]. However, these studies mostly involve small sample sizes.

Developed by Goldberg in the 1970s, the general health questionnaire (GHQ) is a reliable measure of mental health. Moreover, among other versions of GHQ, the GHQ-12 is a self-reported questionnaire that includes 12 items, each of which is assessed with a Likert scale (23). The psychometric properties of this questionnaire have been evaluated in a lot of studies (24–28). Moreover, it has been proven that the GHQ-12 has good specificity, reliability, and sensitivity (29, 30). Although the GHQ-12 was initially developed as a unidimensional scale, there are some controversies regarding if GHQ-12 should be used as a unidimensional scale or a multidimensional structure. Between two or three factor models, there is a lot of empirical support behind a three-factor model of the GHQ-12 (31–35), which include GHQ-12A (social dysfunction and anhedonia; six items), GHQ-12B (depression and anxiety; four items), and GHQ-12C (loss of confidence; two items). A typical argument made that favors the use of the unidimensional model of the GHQ-12 rather than the factor solution is that there is a high correlation between these factors. For instance, the correlation between the 3 factors varied from 0.72 to 0.84 in Padrón et al. (33), from 0.76 to 0.89 in Campbell and Knowles (32), and from 0.83 to 0.90 in Gao et al. (35). However, recent studies using simulated data has proven the imposition of a simple structure may artificially inflate correlations between modeled factors [e.g., (36)]. Thus, as suggested by Griffith and Jones (37), “taking these correlations as justification for unidimensionality risks a self-fulfilling prophecy of simplicity begetting simplicity.” Given these controversies, the current study considers both the unidimensional and multidimensional structure of the GHQ-12.

Thus, although previous studies have shed light on the associations between diabetes and various types of mental health issues with a focus on depression and anxiety, much less is known about how diabetes is associated with other dimensions of mental health such as social dysfunction and anhedonia and loss of confidence in a large nationally representative survey from the United Kingdom. The aim of the current study is to investigate how diabetes is related to general mental health and dimensions of mental health. The current study hypothesized that the GHQ-12 has three underlying factors labeled as GHQ-12A (social dysfunction and anhedonia; six items), GHQ-12B (depression and anxiety; four items), and GHQ-12C (loss of confidence; two items). Moreover, diabetes patients are expected to have worse general mental health and dimensions of mental health.

## Methods

### Data

This study used data from Understanding Society: the UK Household Longitudinal Study (UKHLS), which has been collecting annual information from the original sample of UK households since 1991 [when it was previously known as The British Household Panel Study (BHPS)]. This data set is publicly available at https://www.understandingsociety.ac.uk. The current study used data in Wave 1, which was collected between 2009 and 2010 (38). Participants received written informed consent before participating in the study. Age and sex-matched healthy controls were randomly selected from these participants. Among them, there were 2,255 diabetes patients with a mean age of 60.29 (SD = 14.78) years old with 53.35% males and 14,585 age and sex-matched participants with a mean age of 60.47 (SD = 10.93) years old with 53.40% males who indicated that they were not clinically diagnosed with diabetes.

### Measures

#### Diabetes

Participants answered the question “Has a doctor or other health professional ever told you that you have any of these conditions? Diabetes” to indicate if they have diabetes. Self-reported diabetes is a valid measure of diabetes status in various countries (39–45). However, the specific type of diabetes was not assessed in this sample.

#### Mental health

Mental health was measured using the GHQ-12 (23). The GHQ-12 used the Likert method of scoring ranges from 0 (“Not at all”) to 3 (“Much more than usual”). A summary score across all the 12 items was used to represent general mental health. A higher score means worse mental health. For the purpose of the factor analysis, the GHQ-12 was scored from 1 (“Not at all”) to 4 (“Much more than usual”).

#### Demographic controls

Demographic controls in the model include age (continuous), sex (male = 1 vs. female = 2), monthly income (continuous), highest educational qualification (college = 1 or below college = 2), legal marital status (single = 1 vs. married = 2), and residence (urban = 1 vs. rural = 2).

### Analysis

#### Factor model

A confirmatory factor analysis (CFA) with oblique rotation was applied to the GHQ-12 dataset on MATLAB 2018a with native MATLAB function with a pre-specified number of factors of 3. The three factors are expected to be GHQ-12A (social dysfunction and anhedonia; 6 items), GHQ-12B (depression and anxiety; 4 items), and GHQ-12C (loss of confidence; 2 items). Both the GHQ-12 summary score and factor scores are standardized (mean = 0, std = 1).

#### Linear model

First, a general linear model was constructed by taking demographics from participants without diabetes as predictors and GHQ-12 summary score and three-factor scores underlying the GHQ-12 as the predicted variables respectively. Second, demographic data from participants without diabetes were included in the model as predictors to forecast what GHQ-12 summary score and 3-factor scores underlying the GHQ-12 might be predicted if they have not been clinically diagnosed with diabetes. Finally, a one-sample *t*-test was performed to evaluate if diabetes patients had a higher or lower real GHQ-12 summary score and 3-factor scores underlying the GHQ-12 than predicted. This approach is more advantageous than paired-sample *t*-tests because it can control for demographic confounders.

## Results

The factor analysis yielded three interpretable factors including GHQ-12A (social dysfunction and anhedonia; six items), GHQ-12B (depression and anxiety; four items), and GHQ-12C (loss of confidence; two items). The loadings of these items can be found in Table 1.

**Table 1:** The factor loadings for the three-factor structure of the GHQ-12.

**GHQ-12 items**	**GHQ-12A (social dysfunction and anhedonia; six items)**	**GHQ-12B (depression and anxiety; four items)**	**GHQ-12C (loss of confidence; two items)**
Concentration	**0.5733**	0.1994	−0.1149
Loss of sleep	0.0145	**0.6809**	0.0204
Playing a useful role	**0.6082**	−0.1744	0.1296
Constantly under strain	**0.7411**	−0.1277	−0.0156
Problem overcoming difficulties	−0.0298	**0.8636**	−0.0800
Unhappy or depressed	0.0775	**0.5012**	0.1961
Losing confidence	**0.5747**	0.2341	−0.1211
Believe worthless	**0.6886**	−0.0510	0.0417
General happiness	0.0071	**0.5333**	0.3435
Capable of making decisions	0.0098	0.1650	**0.7207**
Ability to face problems	0.0906	−0.0077	**0.7346**
Enjoy day-to-day activities	**0.4870**	0.1219	0.1188

**Figure 1:**
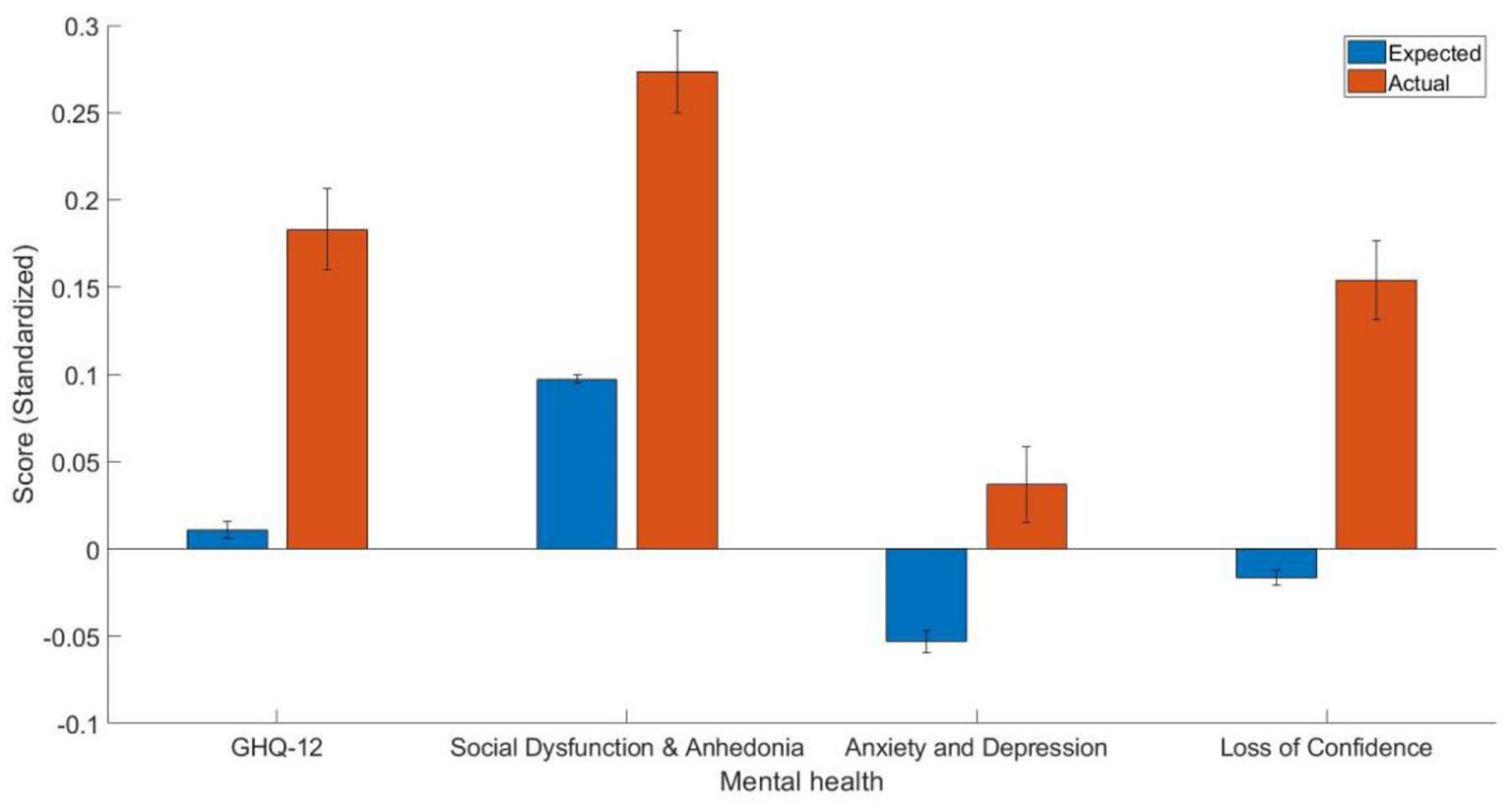
The mean and standard error of the estimated GHQ-12 summary score and three-factor scores (standardized).

The estimate of the predictors in the generalized linear models trained on people without diabetes can be found in Table 2. The current study found that diabetes patients have worse overall mental health as indicated by the GHQ-12 summary score [*t*_(2, 254)_ = 7.49, *p* < 0.001, Cohen's *d* = 0.17, 95% CI (0.13, 0.22)], GHQ-12A [*t*_(2, 254)_ = 7.46, *p* < 0.001, Cohen's *d* = 0.18, 95% CI (0.13, 0.22)], GHQ-12B [*t*_(2, 254)_ = 4.28, *p* < 0.001, Cohen's *d* = 0.09, 95% CI (0.05, 0.13)], GHQ-12C [*t*_(2, 254)_=7.68, *p* < 0.001, Cohen's *d* = 0.17, 95% CI (0.13, 0.21)]. The mean and standard error of predicted and actual standardized scores can be found in Figure 1.

**Table 2:** The estimates (*b*) of linear models trained based on demographic predictors.

	**GHQ-12**	**GHQ-12A**	**GHQ-12B**	**GHQ-12C**
Age	−0.01^***^	0.00	−0.02^***^	−0.01^***^
Sex	0.09^***^	0.02^***^	0.15^**^	0.06^***^
Monthly income	0.00^***^	0.00^***^	0.00^***^	0.00^***^
Highest educational qualification	−0.08^***^	−0.09^***^	−0.04	−0.08^***^
Legal marital status	−0.21^***^	−0.16^***^	−0.16 ^***^	−0.22^***^
Residence	−0.08^***^	−0.06^**^	−0.09^***^	−0.03

All numbers are rounded up to two digits.

*p < 0.05;

** *p* < 0.01;

*** *p* < 0.001.

## Discussion

The aim of the current study was to investigate how diabetes is associated with mental health in general and the dimensions of mental health. The current study predicted that diabetes patients have worse mental health in general and dimensions of mental health. Indeed, the results obtained from the train-and-test approach are consistent with the hypothesis that general mental health and dimensions of mental health are affected by diabetes.

In the current study, the factor analysis yielded 3 factors including GHQ-12A (social dysfunction and anhedonia; six items), GHQ-12B (depression and anxiety; four items), and GHQ-12C (loss of confidence; two items). The 3-factor structure solution found in the current study is largely consistent with previous studies that identified three factors in GhQ-12 (29, 42, 46). Moreover, as shown in Table 1, the factor loadings were represented to be high in the current study.

Importantly, the main findings of the current study were that diabetes patients have worse mental health than controls, which is largely consistent with the literature. Regarding the negative association between diabetes and social dysfunction and anhedonia, previous studies have found that anhedonia was associated with an increased chance of suboptimal glycemic control (HbA1c ≥ 7%) in patients with type 2 diabetes [(47), and see (48) for a review]. Diabetes patients also had depression and anxiety problems in the current study, which is largely consistent with the literature [e.g., (7, 8)]. For instance, it has been shown that clinically relevant depressive symptoms among people with diabetes are ~30% (7, 8). Moreover, clinically diagnosed diabetes but not undiagnosed diabetes was associated with a doubling of the prescriptions for antidepressants, which is consistent with the hypothesis that the associations between diabetes and depression may be attributable to factors that relate to diabetes management (49). Individuals with depression may have a 40–60% increased risk of developing type 2 diabetes (49, 50). Furthermore, the co-occurrence of depression and diabetes is worse than when each illness occurs separately (5). Depression in people with diabetes may amplify the symptom burden by a factor of around 4 (51). Depression in diabetes patients lasts longer and has a higher likelihood to reoccur compared to people without diabetes (52). Major depressive disorder is also associated with underdiagnosed in people with diabetes (53). On the other hand, anxiety is a typical comorbid with depressive symptoms (54). Grigsby et al. (11) estimated that 14% of patients with diabetes suffer from a generalized anxiety disorder. Moreover, this number doubled for patients who experience subclinical anxiety disorder and tripled for patients who had at least some anxiety symptoms (11). Anxiety disorders were also found in one-third of people with type 2 diabetes and serious mental illness and were also associated with increased depressive symptoms and decreased level of function (55). In addition, long-term anxiety is positively related to the risk of developing type 2 diabetes (12). The finding that diabetes patients experience more loss of confidence is consistent with the notion that chronic diseases including diabetes are associated with loss of confidence [e.g., (56)]. This finding implies that improving confidence in patients is important because it may lead to better outcomes (57). Moreover, the effect size as indicated by Cohen's d is smaller for depression and anxiety may indicate that diabetes patients are more affected in other dimensions of mental health, so research in this area should not focus on anxiety and depression alone in diabetes patients.

In conclusion, the present study confirmed the three-factor solution of the GHQ-12 and applied it to a sample of participants who indicated that they had diabetes. Although there are strengths of the current study including a large sample size and a train-and-test approach that takes demographics into account, there are also some limitations. First, although participants indicated that they had diabetes, it remains unclear what types of diabetes (e.g., type 1 vs. type 2) they had. Future studies should ask about specific diabetes that patients might have and how they relate differently to mental health. Second, given the nature of the current study is cross-sectional, it remains unclear if diabetes causes people to have worse mental health or if worse mental health causes people to have diabetes. Future studies should adopt longitudinal approaches in order to establish causality. Third, the current study was largely based on self-reported measures, which could cause self-reported bias. Future studies should use a more objective assessment of diabetes and mental health. Finally, the data was quite old, which may be less informative today. Future studies should use more recent data to study such associations.

## Data availability statement

Publicly available datasets were analyzed in this study. This data can be found here: https://www.understandingsociety.ac.uk.

## Ethics statement

The studies involving human participants were reviewed and approved by University of Essex. The patients/participants provided their written informed consent to participate in this study.

## Author contributions

WK: conceptualization, data curation, formal analysis, investigation, methodology, resources, software, writing—original draft, and writing—review and editing.

## References

[1] Snoek FJ Kersch NY Eldrup E Harman-Boehm I Hermanns N Kokoszka A. Monitoring of individual needs in diabetes (MIND): baseline data from the cross-national Diabetes Attitudes, Wishes, and Needs (DAWN) MIND study. Diabetes Care. (2011) 34:601–3. 10.2337/dc10-1552 21266654 PMC3041189

[2] Robinson DJ Coons M Haensel H Vallis M Yale JF. Diabetes and mental health. Can J Diabetes. (2018) 42:S130–41. 10.1016/j.jcjd.2017.10.031 29650085

[3] Hagger V Hendrieckx C Sturt J Skinner TC. Diabetes distress among adolescents with type 1 diabetes: a systematic review. Curr Diab Rep. (2016) 16:9. 10.1007/s11892-015-0694-2 26748793

[4] Polonsky WH Fisher L Earles J Dudl RJ Lees J Mullan J. Assessing psychosocial distress in diabetes: development of the diabetes distress scale. Diabetes Care. (2005) 28:626–31. 10.2337/diacare.28.3.626 15735199

[5] Polonsky WH Hajos TR Dain MP. Are patients with type 2 diabetes reluctant to start insulin therapy? An examination of the scope and underpinnings of psychological insulin resistance in a large, international population. Curr Med Res Opin. (2011) 27:1169–74. 10.1185/03007995.2011.573623 21469914

[6] Egede LE Nietert PJ Zheng D. Depression and all-cause and coronary heart disease mortality among adults with and without diabetes. Diabetes Care. (2005) 28:1339–45. 10.2337/diacare.28.6.1339 15920049

[7] Anderson RJ Freedland KE Clouse RE. The prevalence of comorbid depression in adults with diabetes: a meta-analysis. Diabetes Care. (2001) 24:1069–78. 10.2337/diacare.24.6.1069 11375373

[8] Ali S Stone MA Peters JL Davies MJ. The prevalence of co-morbid depression in adults with type 2 diabetes: a systematic review and meta-analysis. Diabetes Med. (2006) 23:1165–73. 10.1111/j.1464-5491.2006.01943.x 17054590

[9] Barnard KD Skinner TC Peveler R. The prevalence of co-morbid depression in adults with type 1 diabetes: systematic literature review. Diabetes Med. (2006) 23:445–8. 10.1111/j.1464-5491.2006.01814.x 16620276

[10] Golden SH Lazo M Carnethon M Bertoni AG Schreiner PJ Roux AVD. Examining a bidirectional association between depressive symptoms and diabetes. JAMA. (2008) 299:2751–9. 10.1001/jama.299.23.2751 18560002 PMC2648841

[11] Grigsby AB Anderson RJ Freedland KE Clouse RE. Prevalence of anxiety in adults with diabetes: a systematic review. J Psychosom Res. (2002) 53:1053–60. 10.1016/S0022-3999(02)00417-8 12479986

[12] Hasan SS Clavarino AM Mamun AA. Anxiety symptoms and the risk of diabetes mellitus in Australian women: evidence from 21-year follow-up. Public Health. (2016) 130:1–8. 10.1016/j.puhe.2015.07.022 26321179

[13] Calkin CV Ruzickova M Uher R Hajek T Slaney CM Garnham JS. Insulin resistance and outcome in bipolar disorder. Br J Psychiatry. (2015) 206:52–7. 10.1192/bjp.bp.114.152850 25323142

[14] Mansur RB Rizzo LB Santos CM Asevedo E Cunha GR Noto MN. Impaired glucose metabolism moderates the course of illness in bipolar disorder. J Affect Disord. (2016) 195:57–62. 10.1016/j.jad.2016.02.002 26866976

[15] Lieberman JA Stroup TS McEvoy JP Swartz MS Rosenheck RA Perkins DO. Effectiveness of antipsychotic drugs in patients with chronic schizophrenia. N Engl J Med. (2005) 353:1209–23. 10.1056/NEJMoa051688 16172203

[16] Hackett RA Lazzarino AI Carvalho LA Hamer M Steptoe A. Hostility and physiological responses to acute stress in people with type 2 diabetes. Psychosom Med. (2015) 77:458–66. 10.1097/PSY.0000000000000172 25886832 PMC4431675

[17] Nefs G Speight J Pouwer F Pop V Bot M. Type D personality, suboptimal health behaviors and emotional distress in adults with diabetes: results from Diabetes MILESThe Netherlands. Diabetes Res Clin Pract. (2015) 108:94–105. 10.1016/j.diabres.2015.01.015 25686507

[18] Farr OM Ko BJ Joung KE Zaichenko L Usher N Tsoukas M. Posttraumatic stress disorder, alone or additively with early life adversity, is associated with obesity and cardiometabolic risk. Nutr Metab Cardiovasc Dis. (2015) 25:479–88. 10.1016/j.numecd.2015.01.007 25770759 PMC4404181

[19] Kelly SJ Ismail M. Stress and type 2 diabetes: a review of how stress contributes to the development of type 2 diabetes. Annu Rev Public Health. (2015) 36:441–62. 10.1146/annurev-publhealth-031914-122921 25581145

[20] Colton PA Olmsted MP Daneman D Farquhar JC Wong H Muskat S. Eating disorders in girls and women with type 1 diabetes: a longitudinal study of prevalence, onset, remission, and recurrence. Diabetes Care. (2015) 38:1212–7. 10.2337/dc14-2646 25887359

[21] Jones JM Lawson ML Daneman D Olmsted MP. Eating disorders in adolescent females with and without type 1 diabetes: cross sectional study. BMJ. (2000) 320:1563–6. 10.1136/bmj.320.7249.1563 10845962 PMC27398

[22] Ramos AR Wallace DM Pandi-Perumal SR Williams NJ Castor C Sevick MA. Associations between sleep disturbances and diabetes mellitus among blacks with metabolic syndrome: results from the Metabolic Syndrome Outcome study (MetSO). Ann Med. (2015) 47:233–7. 10.3109/07853890.2015.1015601 25856540 PMC4659349

[23] Goldberg D Williams P. A User's Guide to the General Health Questionnaire. Windsor: NFER-NELSON (1988).

[24] El-Metwally A Javed S Razzak HA Aldossari KK Aldiab A Al-Ghamdi SH. The factor structure of the general health questionnaire (GHQ12) in Saudi Arabia. BMC Health Serv Res. (2018) 18:1–11. 10.1186/s12913-018-3381-6 30071833 PMC6472711

[25] Fernandes HM Vasconcelos-Raposo J. Factorial validity and invariance of the GHQ-12 among clinical and nonclinical samples. Assessment. (2012) 20:219–29. 10.1177/1073191112465768 23118267

[26] Hankins M. The factor structure of the twelve-item general health questionnaire (GHQ-12): the result of negative phrasing? Clin Pract Epidemiol Mental Health. (2008) 4:10. 10.1186/1745-0179-4-10 18435842 PMC2373289

[27] López MPS Dresch V. The 12-item general health questionnaire (GHQ-12): reliability, external validity and factor structure in the Spanish population. Psicothema. (2008) 20:839–43. 18940092

[28] Salama-Younes M Montazeri A Ismaïl A Roncin C. Factor structure and internal consistency of the 12-item general health questionnaire (GHQ-12) and the subjective vitality scale (VS), and the relationship between them: a study from France. Health Qual Life Outcomes. (2009) 7:1. 10.1186/1477-7525-7-22 19265516 PMC2653033

[29] Daradkeh TK Ghubash R. Reliability, validity, and factor structure of the Arabic version of the 12-item general health questionnaire. Psychol Rep. (2001) 89:85–94. 10.2466/pr0.2001.89.1.85 11729557

[30] Endsley P Weobong B Nadkarni A. The psychometric properties of GHQ for detecting common mental disorder among community dwelling men in Goa, India. Asian J Psychiatr. (2017) 28:106–10. 10.1016/j.ajp.2017.03.023 28784361 PMC5565797

[31] Graetz B. Multidimensional properties of the general health questionnaire. Soc Psychiatry Psychiatr Epidemiol. (1991) 26:132–8. 10.1007/BF00782952 1887291

[32] Campbell A Knowles SA. Confirmatory factor analysis of the GHQ12 using a large Australian sample. Eur J Psychol Assess. (2007) 23:2–8. 10.1027/1015-5759.23.1.2

[33] Padrón A Galán I Durbán M Gandarillas A. Confirmatory factor analysis of the general health questionnaire (GHQ-12) in Spanish adolescents. Qual Life Res. (2012) 21:1291–8. 10.1007/s11136-011-0038-x 21997139

[34] Penninkilampi-Kerola V Miettunen J Ebeling H. Health and disability: a comparative assessment of the factor structures and psychometric properties of the GHQ-12 and the GHQ-20 based on data from a Finnish population-based sample. Scand J Psychol. (2006) 47:431–40. 10.1111/j.1467-9450.2006.00551.x 16987212

[35] Gao W Stark D Bennett MI Siegert RJ Murray S. Using the 12-item general health questionnaire to screen psychological distress from survivorship to end-of-life care: dimensionality and item quality. Psychooncology. (2012) 21:954–61. 10.1002/pon.1989 21557386

[36] Marsh HW Morin AJ Parker PD Kaur G. Exploratory structural equation modeling: an integration of the best features of exploratory and confirmatory factor analysis. Annu Rev Clin Psychol. (2014) 10:85–110. 10.1146/annurev-clinpsy-032813-153700 24313568

[37] Griffith GJ Jones K. Understanding the population structure of the GHQ-12: Methodological considerations in dimensionally complex measurement outcomes. Soc Sci Med. (2019) 243:112638. 10.1016/j.socscimed.2019.112638 31665657

[38] University University of Essex Institute for Social and Economic Research. Understanding Society: Waves 1-11, 2009-2020. and Harmonised BHPS: Waves 1-18, 1991–2009, 15th Edition. UK Data Service. SN: 6614 (2022).

[39] Dode MASDO Santos IS. Validity of self-reported gestational diabetes mellitus in the immediate postpartum. Cad Saude Publica. (2009) 25:251–8. 10.1590/S0102-311X2009000200003 19219232

[40] Espelt A Goday A Franch J Borrell C. Validity of self-reported diabetes in health interview surveys for measuring social inequalities in the prevalence of diabetes. J Epidemiol Commun Health. (2012) 66:e15–e15. 10.1136/jech.2010.112698 21502089

[41] Goto A Morita A Goto M Sasaki S Miyachi M Aiba N. Validity of diabetes self-reports in the Saku diabetes study. J Epidemiol. (2013) 2013:JE20120221. 10.2188/jea.JE20120221 23774288 PMC3709549

[42] Martin LM Leff M Calonge N Garrett C Nelson DE. Validation of self-reported chronic conditions and health services in a managed care population. Am J Prev Med. (2000) 18:215–8. 10.1016/S0749-3797(99)00158-0 10722987

[43] Pastorino S Richards M Hardy R Abington J Wills A Kuh D. Validation of self-reported diagnosis of diabetes in the 1946 British birth cohort. Prim Care Diabetes. (2015) 9:397–400. 10.1016/j.pcd.2014.05.003 25037637 PMC4582042

[44] Schneider AL Pankow JS Heiss G Selvin E. Validity and reliability of self-reported diabetes in the atherosclerosis risk in communities study. Am J Epidemiol. (2012) 176:738–43. 10.1093/aje/kws156 23013620 PMC3571247

[45] Yuan X Liu T Wu L Zou ZY Li C. Validity of self-reported diabetes among middle-aged and older Chinese adults: the China Health and Retirement Longitudinal Study. BMJ Open. (2015) 5:e006633. 10.1136/bmjopen-2014-006633 25872937 PMC4401856

[46] Rajabi G Sheykhshabani SH. Factor structure of the 12-item general health questionnaire. J Educ Psychol. (2009) 3:81–94.

[47] Nefs G Bot M Browne JL Speight J Pouwer F. Diabetes MILES-The Netherlands: rationale, design and sample characteristics of a national survey examining the psychosocial aspects of living with diabetes in Dutch adults. BMC Public Health. (2012) 12:1–11. 10.1186/1471-2458-12-925 23110382 PMC3560187

[48] Carter J Swardfager W. Mood and metabolism: anhedonia as a clinical target in type 2 diabetes. Psychoneuroendocrinology. (2016) 69:123–32. 10.1016/j.psyneuen.2016.04.002 27088371

[49] Mezuk B Johnson-Lawrence V Lee H Rafferty JA Abdou CM Uzogara EE. Is ignorance bliss? Depression, antidepressants, and the diagnosis of prediabetes and type 2 diabetes. Health Psychol. (2013) 32:254–63. 10.1037/a0029014 23437855 PMC3725143

[50] Rotella F Mannucci E. Depression as a risk factor for diabetes: a metaanalysis of longitudinal studies. J Clin Psychiatry. (2013) 74:31–7. 10.4088/JCP.12r07922 23419223

[51] Ludman EJ Katon W Russo J Von Korff M Simon G Ciechanowski P. Depression and diabetes symptom burden. Gen Hosp Psychiatry. (2004) 26:430–6. 10.1016/j.genhosppsych.2004.08.010 15567208

[52] Peyrot M Rubin RR. Persistence of depressive symptoms in diabetic adults. Diabetes Care. (1999) 22:448–52. 10.2337/diacare.22.3.448 10097927

[53] Engum A Mykletun A Midthjell K Holen A. Depression and diabetes: a large population-based study of sociodemographic, lifestyle, and clinical factors associated with depression in type 1 and type 2 diabetes. Diabetes Care. (2005) 28:1904–9. 10.2337/diacare.28.8.1904 16043730

[54] Katon W Lin EH Kroenke K. The association of depression and anxiety with medical symptom burden in patients with chronic medical illness. Gen Hosp Psychiatry. (2007) 29:147–55. 10.1016/j.genhosppsych.2006.11.005 17336664

[55] Bajor LA Gunzler D Einstadter D Thomas C McCormick R Perzynski AT. Associations between comorbid anxiety, diabetes control, and overall medical burden in patients with serious mental illness and diabetes. Int J Psychiatry Med. (2015) 49:309–20. 10.1177/0091217415589307 26060262 PMC4698974

[56] Marshall M Carter B Rose K Brotherton A. Living with type 1 diabetes: perceptions of children and their parents. J Clin Nurs. (2009) 18:1703–10. 10.1111/j.1365-2702.2008.02737.x 19646116

[57] Skelly AH Marshall JR Haughey BP Davis PJ Dunford RG. Self-efficacy and confidence in outcomes as determinants of self-care practices in inner-city, African-American women with non-insulin-dependent diabetes. Diabetes Educ. (1995) 21:38–46. 10.1177/014572179502100107 7835203

